# Basal metabolic rate predicts dementia in community-dwelling older adults: a 5-year longitudinal study

**DOI:** 10.1007/s41999-025-01322-9

**Published:** 2025-10-10

**Authors:** Daiki Yamagiwa, Osamu Katayama, Ryo Yamaguchi, Shoma Akaida, Keitaro Makino, Hiroyuki Shimada

**Affiliations:** 1 Department of Preventive Gerontology, Center for Gerontology and Social Science, National Center for Geriatrics and Gerontology, 7-430 Morioka-cho, Obu, Aichi 474-8511 Japan; 2 Center for Environmental and Health Sciences, Hokkaido University, Sapporo, Hokkaido 060-0812 Japan

**Keywords:** Basal metabolic rate, Community-dwelling older adult, Dementia, Nursing care prevention

## Abstract

**Purpose:**

Although basal metabolic rate (BMR) is reportedly associated with physiological function decline, there is no consensus regarding its relationship with cognitive function. Therefore, we investigated the relationship between BMR and dementia risk over a 5-year period in 2550 of the 2975 older adults who were available for analysis (age 73.2 ± 6.0 years, 59.8% women).

**Methods:**

BMR was calculated using the TANITA, Mifflin–St Jeor, Harris–Benedict, and Cunningham equations. Furthermore, the BMR assessment method developed by the National Institute of Health and Nutrition (NIBIOHN) was used. Participants were classified into quartiles for BMR obtained using each method. Cox proportional hazards regression analysis was performed using the highest quartile (Q4) of each BMR to evaluate the association between BMR and dementia risk. Additionally, differences in the performance of each formula in predicting dementia risk were examined using the time-dependent receiver operating characteristic curve.

**Results:**

Participants in the lowest BMR quartile (Q1) had a higher risk of dementia than those in Q4 [TANITA BMR: hazard ratio (HR) = 2.49, *p* < 0.001; Harris–Benedict BMR: HR = 1.70, *p* < 0.05; Mifflin–St. Jeor BMR: HR: 3.83, *p* < 0.001; Cunningham BMR: HR = 2.78, *p* < 0.001; NIBIOHN BMR: HR = 2.98, *p* < 0.001]. The time-dependent ROC of Harris–Benedict BMR demonstrated the highest predictive accuracy among all BMRs calculated (area under the curve = 0.71, *p* < 0.05).

**Conclusion:**

Our results reveal the association of low BMR with increased dementia risk, suggesting its potential as an early predictive biomarker, and demonstrate the usefulness of the Harris–Benedict equation for predicting dementia risk.

**Supplementary Information:**

The online version contains supplementary material available at 10.1007/s41999-025-01322-9.

## Key summary points

**Aim:**

To investigate the association between basal metabolic rate (BMR) and the risk of dementia over 5 years in 3108 older adults.

**Findings:**

Participants in the lowest BMR quartile had a significantly higher risk of developing dementia compared to those in the highest quartile. Among the BMR estimation formulas, the Harris–Benedict equation showed the highest predictive accuracy based on time-dependent ROC analysis.

**Message:**

Low BMR may serve as an early biomarker for dementia risk, with the Harris–Benedict formula offering particularly useful predictive utility.

**Supplementary Information:**

The online version contains supplementary material available at 10.1007/s41999-025-01322-9.

## Introduction

The risk of dementia, a neurodegenerative disease, increases with age. The symptoms of dementia, such as memory loss and impaired judgment, considerably impair quality of life [1, 2]. In the world, the number of patients with dementia is rapidly increasing owing to the aging population, and the associated burden of caregiving and medical expenses has become a major social issue [3, 4]. Early detection of dementia and establishment of preventive strategies are crucial to dementia prevention [5–7]. In particular, elucidating lifestyle-related and physiological factors associated with dementia onset is crucial to the development of effective preventive interventions [8].

Basal metabolic rate (BMR) refers to the minimum amount of energy required to sustain life and accounts for approximately 60% of resting energy expenditure [9]. It is necessary for fundamental physiological functions such as breathing, blood circulation, thermoregulation, and cellular metabolic activity [10]. BMR varies among individuals and is influenced by factors such as age, weight, muscle mass, lifestyle, and health status [11]. Although BMR is largely determined by muscle mass, at the cellular level energy metabolism depends on Adenosine triphosphate (ATP) production, with mitochondrial oxidative phosphorylation playing a key role [12–14]. A decline in BMR is associated with reduced mitochondrial ATP production and decreased energy demand due to muscle mass loss [15, 16]. Additionally, reduced thyroid hormone levels and other regulatory factors can impair metabolic function by decreasing cellular energy supply [17, 18]. These studies have reported that decreases in BMR and muscle mass are associated with neurodegeneration and cognitive decline, but the causal relationship remains unclear. It has also been suggested that neurodegeneration may itself lead to muscle loss and metabolic decline, highlighting the bidirectional interaction between the brain and muscles. Therefore, maintaining appropriate exercise habits and nutritional balance is crucial for preventing a decline in basal metabolism and supporting overall health.

Recent studies have explored the relationship between BMR and cognitive function in neurodegenerative diseases. A significant positive correlation between BMR and cognitive scores has been reported, suggesting that higher BMR may protect against cognitive decline, particularly in Alzheimer’s disease (AD) and Parkinson's dementia [19, 20]. This protective effect has also been supported by evidence from Mendelian randomization, indicating a bidirectional causal relationship between BMR and AD [21]. In contrast, no evidence of lower resting metabolic rates in AD patients was reported in an earlier study [22]. Overall, the findings remain inconsistent, and importantly, all previous investigations have been cross-sectional; thus, it is still unclear how BMR influences the future onset of dementia. Moreover, although several equations exist for calculating BMR, including the Harris–Benedict, Mifflin–St. Jeor and Cunningham equations, their impact on dementia risk prediction remains uncertain.

This study aims to investigate the effect of BMR on the onset of dementia during 5 years in community-dwelling older Japanese adults, and to further identify a calculation formula for BMR that is highly accurate in predicting the onset of dementia. We hypothesized that a decrease in BMR increases the risk of developing dementia 5 years later. If this hypothesis is supported, it will demonstrate that BMR is a more convenient predictor of dementia. Furthermore, identifying the optimal calculation equation for measuring BMR is expected to provide a more accurate indicator for future dementia prevention, leading to improved effectiveness of preventive interventions.

## Methods

### Participants

**Fig. 1:**
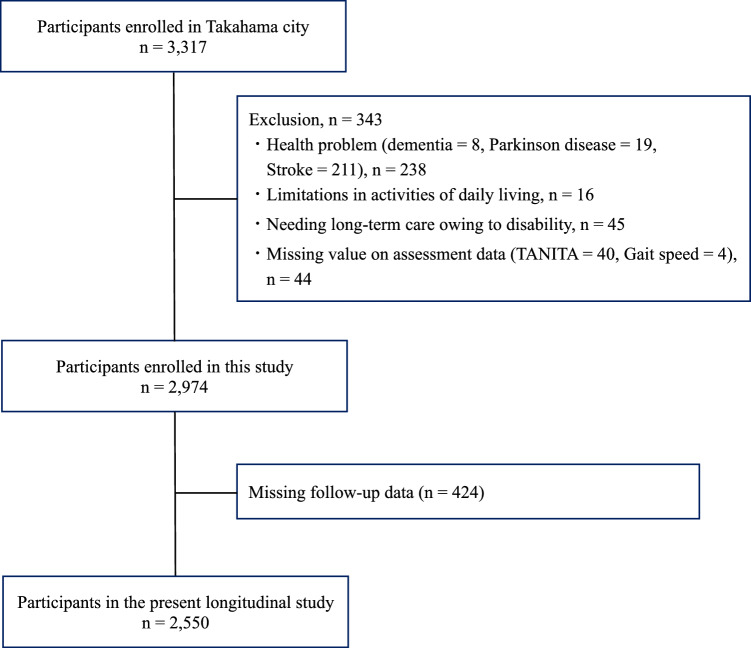
Participant flow diagram

In this community-based study, we analyzed data from 3317 community‐dwelling older Japanese adults. Participants were recruited from a sub-cohort of the National Center for Geriatrics and Gerontology–Study of Geriatric Syndromes (NCGG-SGS) study, a large-scale cohort study aimed at identifying risk factors for geriatric syndromes and developing effective management strategies [23]. To be eligible, individuals had to reside in Takahama City, Aichi Prefecture, Japan, and be at least 65 years old at baseline. Data collection involved structured face-to-face interviews to obtain demographic and medical information, self-reported questionnaires on lifestyle and living conditions, as well as physical and cognitive function assessments. These evaluations were carried out by trained healthcare professionals and research staff. The exclusion criteria were as follows: (1) a prior diagnosis of dementia, Parkinson’s disease, or stroke (*n* = 238); (2) difficulty in performing basic activities of daily living (*n* = 16); (3) enrollment in the long-term care insurance (LTCI) system at baseline (*n* = 45); and (4) incomplete assessment data (*n* = 44). The final baseline cohort comprised 2974 individuals. The follow-up phase, in which we tracked dementia onset and functional decline, involved 2550 participants, after excluding 424 individuals with missing follow-up data (Fig. 1). Baseline assessments were conducted between September 2015 and June 2016, with a 5-year follow-up period thereafter.

This study was conducted in accordance with the ethical principles of the Declaration of Helsinki and was approved by the Institutional Review Board of the National Center for Geriatrics and Gerontology (approval number: 1440-8, approved on July 29, 2025). Before participation, all individuals provided written informed consent. The research followed the Strengthening the Reporting of Observational Studies in Epidemiology (STROBE) guidelines for reporting observational studies.

### BMR calculation

BMR was estimated using five different equations: the TANITA, Harris–Benedict, Mifflin–St. Jeor, Cunningham, and Japanese Dietary Reference Intakes [JDRI; National Institute of Biomedical Innovation, Health, and Nutrition (NIBIOHN)] equations. Each calculation equation employs different parameters and assumptions for calculating energy expenditure.

### TANITA BMR

TANITA BMR [kcal/day] is determined using bioelectrical impedance analysis [24], which evaluates body composition by measuring electrical resistance through tissues. In this study, the tests were performed using a multifrequency bioelectrical impedance analyzer (MC-980A, TANITA, Tokyo, Japan). Unlike traditional equations based solely on demographic factors, this calculation equation accounts for individual variations in muscle and fat masses, providing a personalized metabolic rate estimation [25].

### Harris–Benedict BMR revised 1984

Proposed in 1984 by Roza and Shizgal, the revised Harris–Benedict equation is a widely used predictive model for estimating BMR [26]. It calculates energy expenditure based on weight, height, age, and sex, and has been adjusted to better reflect contemporary populations [27].

$$\begin{aligned} &{\text{Male BMR }}\left[ {{\text{kcal}}/{\text{day}}} \right] \\ &\quad = 88.362{ } + { }\left( {13.397{ } \times {\text{ Weight}}} \right){ } + { }\left( {4.799 \times {\text{Height}}} \right) - \left( {5.677 \times {\text{Age}}} \right)\\ & {\text{Female BMR }}\left[ {{\text{kcal}}/{\text{day}}} \right] \\ & \quad = 447.593 + \left( {9.247{ } \times {\text{ Weight}}} \right){ } + { }\left( {3.098 \times {\text{Height}}} \right) - \left( {4.330 \times {\text{Age}}} \right) \\ \end{aligned}$$

### Mifflin–St Jeor BMR

Introduced in 1990, the Mifflin–St. Jeor equation was designed to provide a more precise estimation of BMR than the Harris–Benedict equation [28, 29]. It remains widely used owing to its improved accuracy and is particularly beneficial in settings where metabolic rate estimation is crucial for nutritional planning and weight management [30].

$$\begin{aligned} & {\text{Male BMR }}\left[ {{\text{kcal}}/{\text{day}}} \right] \\ & \quad = \left( {10{ } \times {\text{ Weight}}} \right){ } + { }\left( {6.25{ } \times {\text{ Height}}} \right){ } - { }\left( {5{ } \times {\text{ Age}}} \right){ } + { }5 \\ & {\text{Female BMR }}\left[ {{\text{kcal}}/{\text{day}}} \right] \\ & \quad = { }\left( {10{ } \times {\text{ Weight}}} \right){ } + { }\left( {6.25{ } \times {\text{ Height}}} \right){ } - { }\left( {5{ } \times {\text{ Age}}} \right){ } - { }161 \\ \end{aligned}$$

### Cunningham BMR

The Cunningham equation incorporates fat-free mass (FFM) as a key variable, making it particularly suitable for individuals with higher muscle mass [31]. In this study, FFM calculated from TANITA was used. As muscle tissue requires more energy than fat tissue, this calculation equation provides a more individualized estimate of BMR [32].

$$\begin{aligned} & {\text{BMR }}\left[ {{\text{kcal}}/{\text{day}}} \right] \\ & \quad = { }500{ } + { }\left( {22{ } \times {\text{ lean body mass}}} \right) \\ \end{aligned}$$

### Japanese dietary reference intakes (JDRI; NIBIOHN) BMR

The NIBIOHN developed a BMR-estimation equation specifically tailored for the Japanese population [33, 34]. This calculation equation is based on extensive data collected from Japanese men and women and considers weight, height, age, and sex, making it particularly relevant for metabolic assessments within this demographic.

$$\begin{aligned} &{\text{Male BMR }}\left[ {{\text{kcal}}/{\text{day}}} \right]\\ &\quad = \left( {\left( {0.1238 + \left( {0.0481 \times {\text{Weight}}} \right) + \left( {0.0234 \times {\text{Height}}} \right) - \left( {0.0138 \times {\text{ Age}}} \right) - { }0.5473} \right)} \right) \\ &\quad\quad \times 1000/4.186 \\ & {\text{Female BMR }}\left[ {{\text{kcal}}/{\text{day}}} \right] \\ &\quad = \left( {\left( {0.1238 + \left( {0.0481 \times {\text{Weight}}} \right) + \left( {0.0234 \times {\text{Height}}} \right) - \left( {0.0138 \times {\text{ Age}}} \right) - { }0.5473 \times 2} \right)} \right)\\ &\quad\quad \times 1000/4.186 \\ \end{aligned}$$

### Follow-up of the incidence of dementia

Dementia incidence was determined using data from the NCGG‐SGS study, based on diagnoses recorded with the corresponding ICD‐10 codes or the Dementia Rating Scale of the LTCI system in Japan [35, 36]. Participants were considered to have developed dementia if they were dementia-free at baseline and received a new diagnosis during the 60-month follow-up period.

Dementia diagnosis was tracked through Japan’s public health insurance system, which covers all individuals aged ≥ 65 years under Employees’ Health Insurance, National Health Insurance, or Later-Stage Medical Care. Monthly follow-ups helped identify new-onset dementia cases, including cases of AD, vascular dementia, frontotemporal dementia, and other subtypes, based on physician diagnoses using the ICD‐10 criteria. The NCGG‐SGS dementia identification approach aligns with the UK Biobank inpatient data [37].

Additionally, dementia was assessed through the LTCI system, a mandatory social insurance program supporting older adults with disabilities [38]. All individuals aged ≥ 65 years are eligible for institutional or community-based services depending on their disability level, determined through a standardized certification process involving a questionnaire and a physician’s assessment [39]. Dementia was identified using the Dementia Rating Scale, which categorizes individuals into six ranks (0, I–IV, M). In this study, dementia onset was defined as rank II or higher, indicating mild to moderate impairment [40].

### Potential confounding factors

The confounding factors measured were those previously identified to potentially affect dementia, including age, sex, height, weight, gait speed, cognitive function, Geriatric Depression Scale (GDS) score, number of medications used, smoking status, education, history of heart disease, hypertension, diabetes disease, and hyperlipidemia [41–45]. Gait speed was measured in seconds over a 2.4‐m distance at a comfortable gait speed. The participants walked for an additional 2‐m distance before and after measurement to ensure consistent speed. Cognitive function was assessed using the Mini-Mental State Examination (MMSE). To assess depressive symptoms in older adults, the 15-item version of the GDS was utilized with higher scores corresponding to a greater presence of depressive symptoms [46]. The other factors were collected using a questionnaire.

### Statistical analysis

Each variable was tested for normality using the Kolmogorov–Smirnov test. All variables did not follow normality, and therefore, nonparametric tests were employed. For potential confounding factors and different BMRs, Kruskal–Wallis and *χ* tests were used to compare baseline characteristics in the dementia group who developed dementia during the follow-up period, the survivor group who did not develop dementia, and the Censored group who were lost to follow-up due to death or emigration. Multiple comparisons were performed using Dunn’s test as a post hoc test.

Survival analysis was performed using Kaplan–Meier curves to estimate the cumulative survival rate for dementia onset by classifying patients based on the BMR into four groups by quartile, and differences between groups were examined using the log-rank test. Cox proportional hazards regression analysis was used to calculate the hazard ratio (HR) and 95% confidence interval (CI) for new dementia onset with the fourth quartile (highest BMR) as the reference. The Cox regression model was adjusted for potential confounders such as age, sex, height, weight, gait speed, MMSE, GDS score, education, number of medications, smoking status, heart disease, diabetes, hypertension, and hyperlipidemia. To address potential collinearity, sensitivity analyses excluding covariates that are components of the BMR equations (age, height, and weight) were also performed.

Because death is a competing risk in an elderly cohort, Fine–Gray competing risk regression was additionally conducted to estimate subdistribution hazard ratios (SHR) and to plot cumulative incidence functions of dementia across BMR quartiles. For dose–response analyses, each BMR equation was treated as a continuous variable. Sex-stratified Cox models were performed to estimate hazard ratios per 100 kcal/day increase. In addition, restricted cubic spline (RCS) models with three knots were additionally fitted at 1 kcal/day increments to assess potential non-linear associations between BMR and dementia risk.

To verify predictive performance, time-dependent receiver operating characteristic (time-dependent ROC) analysis was conducted, with a maximum follow-up of 60 months [47, 48]. We calculated cumulative/dynamic AUCs at 15, 30, 45, and 60 months for each BMR equation. Pairwise statistical comparisons of AUCs between equations were performed using the DeLong test. Internal validation was conducted via bootstrap resampling (1000 iterations) to obtain optimism-corrected AUCs. Calibration was assessed using calibration plots comparing predicted and observed risks at 5 years.

All statistical analyses were conducted using SPSS Statistics version 28 (IBM Inc., Tokyo, Japan) and R version 4.4.2 (R Foundation for Statistical Computing, Vienna, Austria). For analyses, we used the following R packages: survival (Cox regression, Kaplan–Meier), cmprsk (Fine–Gray competing risk), rms (restricted cubic spline models, calibration), timeROC (time-dependent ROC analysis), and pROC (AUC comparisons). Statistical significance was defined as *p* < 0.05.

## Results

In this study, the incidence rate of dementia was 21.0 per 1000 person-years (95% CI 18.6–23.7), and that of death was 11.8 per 1000 person-years (95% CI 10.1–13.9). The dementia-onset group exhibited characteristics such as older age, a higher proportion of female patients, lower body weight, a higher prevalence of heart disease, slower gait speed, and a greater number of medications taken than the survival group (Table 1). Additionally, their BMR was significantly lower across all estimation equations, including the Mifflin–St. Jeor, Harris–Benedict, Cunningham, and NIBIOHN. On the contrary, the censored group was characterized by a higher prevalence of diabetes and a greater proportion of smokers. However, their BMR was comparable to that of the survival group, without a significant reduction observed in the dementia-onset group.

**Table 1:** Baseline characteristics of participants by follow-up status

	Survive	Dementia	Censored	*p*-value
	*n* = 2026	*n* = 268	*n* = 251	
Age, year	73.2 ± 5.7	75.1 ± 6.2^*^	75.4 ± 6.1^*^^,$^	< 0.001
Sex, female, *n* (%)	1219 (60.0)	179 (66.5)^*^	123 (49.0)^*^^,$^	< 0.05
Height, cm	155.3 ± 8.6	150.1 ± 9.3^*^	154.9 ± 8.9^$^	< 0.001
Weight, kg	56.8 ± 9.9	52.4 ± 10.4^*^	56.6 ± 11.5^$^	< 0.001
Medical history				
Heart disease, *n* (%)	335 (16.5)	49 (18.2)	46 (18.3)	0.264
Hypertension, *n* (%)	1022 (50.3)	150 (55.8)^*^	143 (57.0)^*^	< 0.05
Diabetes disease, *n* (%)	267 (13.2)	42 (15.6)^*^	51 (20.3)^*^^,$^	0.155
Hyperlipidemia, *n* (%)	652 (32.1)	74 (27.5)	69 (27.5)	0.071
Medication	2.9 ± 2.6	4.2 ± 3.0^*^	3.5 ± 2.8^*^^,$^	< 0.001
Smoking	161 (8.0)	23 (8.6)	30 (12.0)^*^^,$^	< 0.05
Gait speed, m/s	1.1 ± 0.2	0.8 ± 0.2^*^	1.0 ± 0.2^*^^,$^	< 0.001
MMSE, score	27.2 ± 2.4	25.1 ± 3.3^*^	26.1 ± 3.2^*^^,$^	< 0.001
GDS, score	2.8 ± 2.6	3.0 ± 0.2^*^	3.5 ± 3.0^*^^,$^	< 0.001
Education, year	11.0 ± 2.3	10.0 ± 2.3^*^	10.4 ± 2.3^*^	< 0.001
TANITA BMR, kcal/day	1114.1 ± 186.3	1012.1 ± 175.8^*^	1109.9 ± 206.6^$^	< 0.001
Mifflin–St Jeor BMR, kcal/day	1077.5 ± 208.5	955.8 ± 211.6^*^	1077.4 ± 224.8^$^	< 0.001
Harris–Benedict BMR, kcal/day	1158.3 ± 144.3	1057.9 ± 141.8^*^	1140.9 ± 170.0^$^	< 0.001
Cunningham BMR, kcal/day	1373.7 ± 168.3	1291.9 ± 156.5^*^	1376.0 ± 181.3^$^	< 0.001
NIBIOHN BMR, kcal/day	1099.4 ± 199.9	988.8 ± 204.5^*^	1099.7 ± 217.9^$^	< 0.001

BMR, basal metabolic rate; MMSE, mini-mental state examination; GDS, geriatric depression scale; NIBIOHN, National Institutes Of Biomedical Innovation, Health And Nutrition

* versus survive; $ versus dementia

**Fig. 2:**
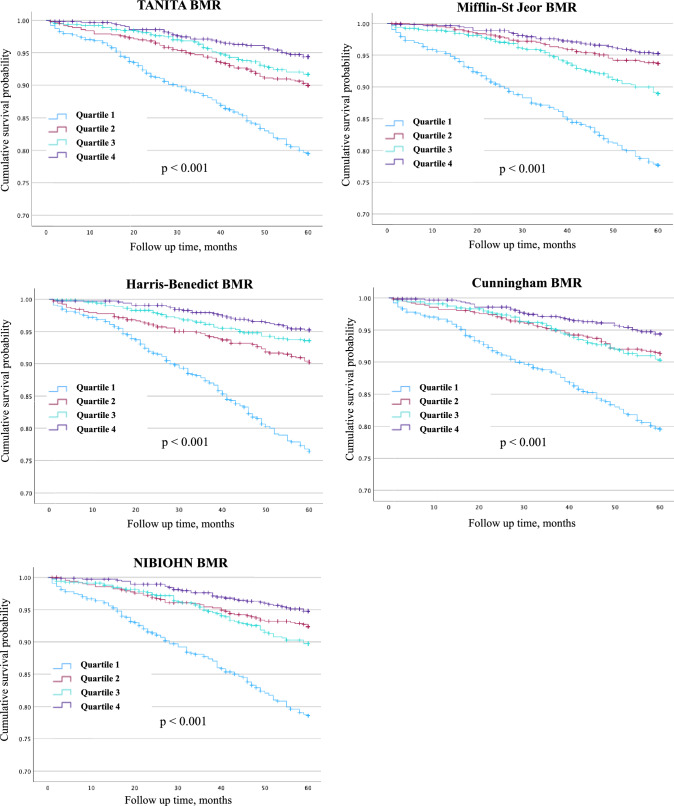
Kaplan–Meier curve analysis results for each BMR calculation equation. The vertical axis shows cumulative survival rate and horizontal axis shows the follow-up time. Light blue indicates the first quartile, red indicates the second quartile, light green indicates the third quartile, and purple indicates the fourth quartile

The quartile ranges for each BMR calculation formula are shown in Supplemental Table S1. The Kaplan–Meier curves showed significant differences in dementia-free survival across the BMR quartiles (log-rank *p* < 0.001) (Fig. 2). In the multivariable Cox regression analyses (Table 2), participants in the lowest quartile (Q1) consistently exhibited a significantly higher risk of dementia onset compared with those in the highest quartile (Q4). This pattern was observed across all five BMR calculation formulas, although the magnitude of the hazard ratios varied: for example, Q1 risk ranged from 1.7-fold higher with the Harris–Benedict formula to nearly fourfold higher with the Mifflin–St. Jeor formula. Sensitivity analyses excluding age, height, and weight from the covariate set yielded similar results, suggesting that multicollinearity did not materially affect the findings (Supplementary Table S2).

**Table 2:** Hazard ratios for dementia incidence by quartiles of five different indicators (full model)

Predictor	Quartile	HR (95% CI)	*p*-value
TANITA BMR	Q4	*Reference*	
	Q3	1.36 (0.86–2.14)	0.19
	Q2	1.7 (1.02–2.82)	< 0.05
	Q1	2.49 (1.46–4.26)	< 0.001
Mifflin–St Jeor BMR	Q4	*Reference*	
	Q3	1.92 (1.21–3.05)	< 0.05
	Q2	2.15 (1.09–4.23)	< 0.05
	Q1	3.83 (1.88–7.81)	< 0.001
Harris–Benedict BMR	Q4	*Reference*	
	Q3	1.05 (0.64–1.72)	0.853
	Q2	1.44 (0.88–2.33)	0.145
	Q1	1.79 (1.10–2.92)	< 0.05
Cunningham BMR	Q4	*Reference*	
	Q3	1.51 (0.97–2.34)	0.07
	Q2	2.00 (1.06–3.65)	< 0.05
	Q1	2.78 (1.47–5.22)	< 0.001
NIBIOHN BMR	Q4	*Reference*	
	Q3	1.70 (1.09–2.657)	< 0.05
	Q2	2.03 (1.14–3.64)	< 0.05
	Q1	2.98 (1.61–5.53)	< 0.001

Cox proportional hazards models were adjusted for age, sex, height, weight, gait speed, MMSE, GDS score, education, smoking, heart disease, hypertension, diabetes disease, and hyperlipidemia. The fourth quartile (Q4) was used as the reference category. BMR; Basal Metabolic Rate, HR; Hazard Ratio, CI; Confidence Interval, NIBIOHN; National Institute of Biomedical Innovation, Health, and Nutrition

In a Fine-Gray analysis that considered death as a competing risk, a trend toward a higher dementia risk was observed in lower quartiles for all five BMR calculation formulas. For example, the subdistribution hazard ratio (SHR) between the Q4 and the Q1 was 1.25 (95% CI 1.12–1.29) for the Harris–Benedict BMR. Results for the other four formulas showed a similar trend (Supplemental Table S4). Cumulative incidence curves also confirmed a trend toward a higher dementia risk in the low BMR group (Supplemental Fig. S1). RCS analysis did not reveal a nonlinear relationship between BMR and dementia risk (*p* for non-linearity < 0.05). The spline curve showed a trend toward a gradually increasing dementia risk with decreasing BMR (Supplemental Fig. S2).

**Fig. 3:**
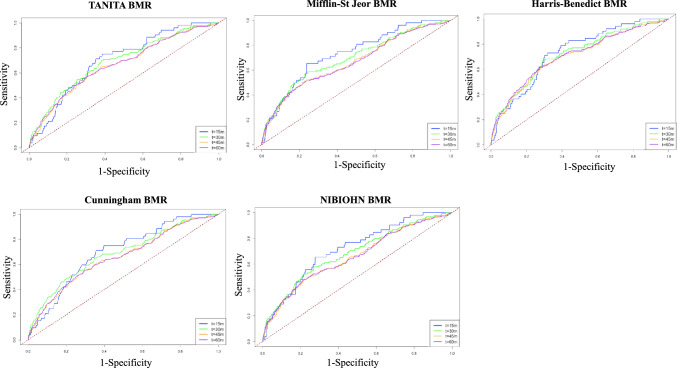
Time-dependent ROC results for each BMR calculation equation. The vertical axis indicates sensitivity and horizontal axis indicates specificity. Blue indicates 15 months of follow-up, yellow-green indicates 30 months of follow-up, orange indicates 45 months of follow-up, and purple indicates 60 months of follow-up

Time-dependent ROC analysis showed that all BMR calculation formulas demonstrated consistent predictive ability at 15 months, but the AUC tended to decrease over time (Table 3 and Fig. 3). Among these, the Harris–Benedict formula maintained significant predictive ability even at 60 months (AUC = 0.71, 95% CI 0.68–0.74, *p* < 0.05), demonstrating the most stable predictive performance. On the other hand, the Mifflin–St. Jeor formula showed a high AUC at 15 months, but lost significance after 30 months. The Cunningham and NIBIOHN formulas also showed no significant predictive ability after 45 months. Although formal statistical comparisons of AUCs did not reveal significant differences between formulas, the Harris–Benedict consistently demonstrated numerically higher and more stable performance. Internal validation using the bootstrap method indicated that optimism was minimal, suggesting limited influence of overfitting (Supplemental Table S5). Furthermore, particularly for the Harris–Benedict and TANITA models, the calibration plots showed good agreement between the observed and predicted values over a 60-month period, confirming relatively good calibration (Supplementary Fig. S3).

**Table 3:** Time-dependent ROC analysis for survival prediction

Indicator	15 months		30 months		45 months		60 months	
	AUC (95% CI)	*p*-value	AUC (95% CI)	*p*-value	AUC (95% CI)	*p*-value	AUC (95% CI)	*p*-value
TANITA BMR	0.72 (0.65–0.78)	< 0.05	0.69 (0.64–0.74)	< 0.05	0.66 (0.62–0.71)	< 0.05	0.66 (0.64–0.69)	< 0.05
Mifflin–St Jeor BMR	0.74 (0.70–0.80)	< 0.05	0.70 (0.65–0.74)	0.08	0.67 (0.63–0.70)	0.16	0.67 (0.63–0.70)	0.33
Harris–Benedict BMR	0.74 (0.70–0.79)	< 0.05	0.72 (0.69–0.76)	< 0.05	0.70 (0.68–0.74)	< 0.05	0.71 (0.68–0.74)	< 0.05
Cunningham BMR	0.71 (0.66–0.77)	< 0.05	0.69 (0.64–0.73)	0.09	0.66 (0.62–0.69)	0.23	0.66 (0.62–0.68)	0.79
NIBIOHN BMR	0.73 (0.69–0.78)	< 0.05	0.68 (0.64–0.73)	< 0.05	0.66 (0.62–0.69)	0.09	0.66 (0.63–0.69)	0.18

Time-dependent ROC calculated the AUC at 15, 30, 45, and 60 months

BMR, basal metabolic rate; AUC, area under the curve, CI, confidence interval, NIBIOHN, national institutes of biomedical innovation, health and nutrition

## Discussion

In this study, we aimed to investigate the effect of BMR on dementia risk over 5 years among community-dwelling Japanese older adults and to identify the optimal BMR calculation equation for prediction. A lower BMR was consistently associated with a higher risk of dementia across all equations. When assessing predictive performance, all equations showed significant accuracy within the first 15 months; however, only the TANITA and Harris–Benedict equations maintained superior accuracy throughout the 5-year period.

A decline in BMR is thought to reflect reduced muscle mass and impaired mitochondrial function, leading to diminished ATP production capacity [49–51]. Lower thyroid hormone levels and reduced whole-body energy metabolism are also considered contributing factors [52]. These metabolic alterations may accelerate neurodegeneration and increase dementia risk [53, 54]. Conversely, neurodegeneration itself can induce muscle loss and metabolic dysfunction, suggesting a bidirectional relationship. In addition, shared pathological mechanisms such as chronic inflammation, hormonal changes, and mitochondrial disorders may underlie both reduced BMR and cognitive decline. In this study, we observed a trend toward a lower risk of dementia in the high BMR group. This finding suggests that maintaining a higher BMR could help preserve cognitive function; however, the causal relationship remains unclear and should be interpreted with caution. Further longitudinal and interventional studies, incorporating bidirectional brain–muscle interactions, are warranted. Furthermore, Fine-Gray competing risk analysis showed that the hazard ratio decreased when death was considered as a competing event, suggesting that the influence of competing risks such as death must be carefully considered when interpreting the association between BMR and dementia risk. Although further research is needed to clarify the relationship between BMR and dementia, this study underscores the potential role of regular exercise and proper nutrition in maintaining BMR, which may contribute to dementia prevention [55].

In a 5-year validation study of dementia onset prediction using multiple BMR equations, no statistically significant differences were observed in AUC comparisons; however, the Harris–Benedict equation consistently showed numerically higher and more stable performance. This tendency was also confirmed by internal validation (bootstrap method), and the influence of overfitting was limited. Furthermore, calibration analysis showed that the Harris–Benedict and TANITA BMR had high consistency between observed and predicted values, enabling highly reliable predictions. Notably, the Harris–Benedict BMR consistently achieved the highest AUC, maintaining strong predictive accuracy of 0.71 even at 60 months. This robustness may reflect its comprehensive inclusion of age, sex, height, and weight, which better capture the metabolic characteristics of older individuals. In contrast, although the Mifflin–St. Jeor equation incorporates the same physiological factors its predictive utility was limited to the first 15 months and lacked long-term capability. This limitation may reflect its design, which is optimized for modern body compositions and suited to point-in-time health assessments rather than extended risk prediction. The TANITA BMR also showed stable performance, but its AUC remained slightly lower than that of the Harris–Benedict BMR. Because it is derived from impedance-based body composition measurements, it may be more vulnerable to intra- and inter-day fluctuations and measurement conditions. Moreover, as it requires large-scale equipment, its practicality is limited unless it provides clearly superior predictive value. Overall, these findings suggest that the Harris–Benedict equation represents the simplest and most effective tool for evaluating dementia risk in older individuals based on BMR.

Although the Harris–Benedict equation is one of the most practical tools for estimating BMR, its clinical utility in dementia risk assessment requires further investigation. Our RCS analysis showed that the risk of dementia significantly increases below 989 kcal/day. These findings suggest that people with relatively low BMR may be at higher risk for subsequent cognitive decline, although a universally accepted threshold has yet to be established. Because BMR can be readily estimated from routinely collected anthropometric measurements, it may serve as a cost-effective screening tool in both community and clinical settings. Furthermore, identifying individuals with low BMR could inform preventive strategies, such as individualized nutritional support and exercise interventions aimed at improving metabolic and cognitive health.

This study demonstrated that BMR influences the risk of developing dementia and highlighted the utility of the Harris–Benedict equation for its assessment. BMR represents a relatively simple indicator for early dementia prediction and the development of preventive strategies. However, several limitations should be acknowledged. First, the study was conducted in a specific region, and similar results may not necessarily be observed in other populations. Second, while different equations were used to estimate BMR, further investigation is needed to assess the accuracy and applicability of each. Third, although some lifestyle factors were considered, this study did not comprehensively account for dietary and exercise habits, which may have influenced the findings. Moreover, several important potential confounders, such as APOE genotype, inflammatory markers, and detailed nutritional status, were not assessed. These factors are associated with both metabolic function and cognitive outcomes, and their omission represents a limitation. Future research should incorporate these variables to provide a more comprehensive and mechanistic understanding of the relationship between BMR and dementia risk. Finally, although we conducted comprehensive comparisons of BMR equations, internal validation, and calibration analyses, we were unable to perform decision curve analysis due to limitations in the available data and software implementation. Nonetheless, the superiority of the Harris–Benedict equation is supported by the other analyses presented.

This study suggested that lower BMR was associated with an increased risk of dementia in older adults and that the Harris–Benedict equation appeared to be the most effective method for estimating BMR in this context. As BMR is a relatively simple and accessible indicator, it may help identify individuals at higher dementia risk and potentially inform preventive strategies. These findings highlight the potential role of metabolic health in cognitive aging and suggest that simple physiological indicators such as BMR could aid early dementia risk stratification.

## Supplementary Information

Below is the link to the electronic supplementary material.

**Supplementary file 1. Fig. 1**. Cumulative incidence of dementia according to basal metabolic rate (BMR) quartiles. The Fine-Gray competing risk model was used, considering death as a competing event. Lines indicate estimated cumulative incidence for each BMR quartile: Quartile 1 (blue), Quartile 2 (green), Quartile 3 (orange), Quartile 4 (red). (DOCX 1396 KB)

**Supplementary file 2. Fig. 2**. Dose–response relationship between BMR and risk of dementia estimated using restricted cubic splines with 3 knots. The solid line represents the estimated hazard ratio, and the shaded area represents the 95% confidence interval. The reference point (HR = 1) is set at the median BMR. (DOCX 935 KB)

**Supplementary file 3. Fig. 3**. Calibration plots for predicted dementia risk at 15, 30, 45, and 60 months using the Fine-Gray competing risk model. The dashed 45° line represents perfect agreement between predicted and observed risk. Solid lines indicate observed risks at each time point: 15 months (blue), 30 months (green), 45 months (orange), and 60 months (purple). (DOCX 755 KB)

Supplementary file 4 (DOCX 19 KB)

Supplementary file 5 (DOCX 22 KB)

Supplementary file 6 (DOCX 19 KB)

Supplementary file 7 (DOCX 22 KB)

Supplementary file 8 (DOCX 20 KB)

## Data Availability

The datasets analyzed in this study were obtained under data use agreements and are not publicly available. They may be available from the corresponding author on reasonable request, subject to approval from the data custodian.
