# Supplementary material for: Basal metabolic rate predicts dementia in community-dwelling older adults: a 5-year longitudinal study
Source: Eur Geriatr Med. 2025 Oct 10;16(6):2181–91. doi: 10.1007/s41999-025-01322-9 (PMC12743684; doi:10.1007/s41999-025-01322-9)
Supplement: Supplementary file 1 — Fig. 1. Cumulative incidence of dementia according to basal metabolic rate (BMR) quartiles. The Fine-Gray competing risk model was used, considering death as a competing event. Lines indicate estimated cumulative incidence for each BMR quartile: Quartile 1 (blue), Quartile 2 (green), Quartile 3 (orange), Quartile 4 (red). (DOCX 1396 KB) [file 41999_2025_1322_MOESM1_ESM.docx]

**Quartile 1**

**Quartile 2**

**Quartile 3**

**Quartile 4**


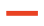

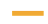

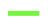

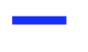


Follow up time, months

Cumulative incidence of dementia

**TANITA BMR**

Cumulative incidence of dementia

Follow up time, months

**Mifflin-St Jeor BMR**

**Quartile 1**

**Quartile 2**

**Quartile 3**

**Quartile 4**


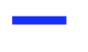

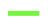

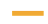

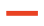


**Cunningham BMR**

Cumulative incidence of dementia

Follow up time, months

**Quartile 1**

**Quartile 2**

**Quartile 3**

**Quartile 4**


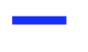

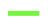

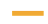

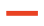


Cumulative incidence of dementia

Follow up time, months

**Harris-Benedict BMR**

**Quartile 1**

**Quartile 2**

**Quartile 3**

**Quartile 4**


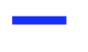

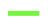

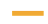

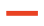


**NIBIOHN BMR**

Cumulative incidence of dementia

Follow up time, months

**Quartile 1**

**Quartile 2**

**Quartile 3**

**Quartile 4**


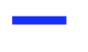

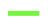

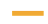

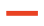


Supplemental Figure 1
