# Supplementary material for: Basal metabolic rate predicts dementia in community-dwelling older adults: a 5-year longitudinal study
Source: Eur Geriatr Med. 2025 Oct 10;16(6):2181–91. doi: 10.1007/s41999-025-01322-9 (PMC12743684; doi:10.1007/s41999-025-01322-9)
Supplement: Supplementary file 2 — Fig. 2. Dose–response relationship between BMR and risk of dementia estimated using restricted cubic splines with 3 knots. The solid line represents the estimated hazard ratio, and the shaded area represents the 95% confidence interval. The reference point (HR = 1) is set at the median BMR. (DOCX 935 KB) [file 41999_2025_1322_MOESM2_ESM.docx]

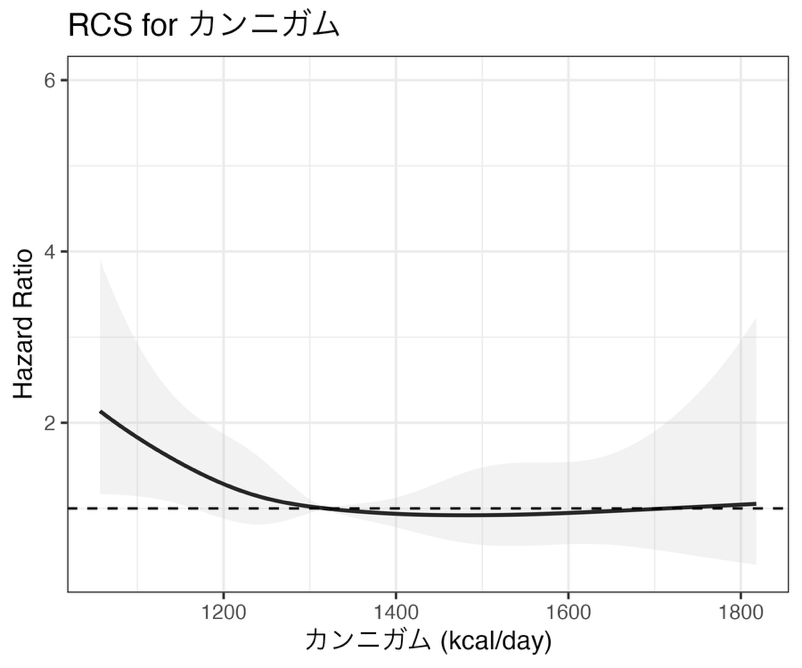


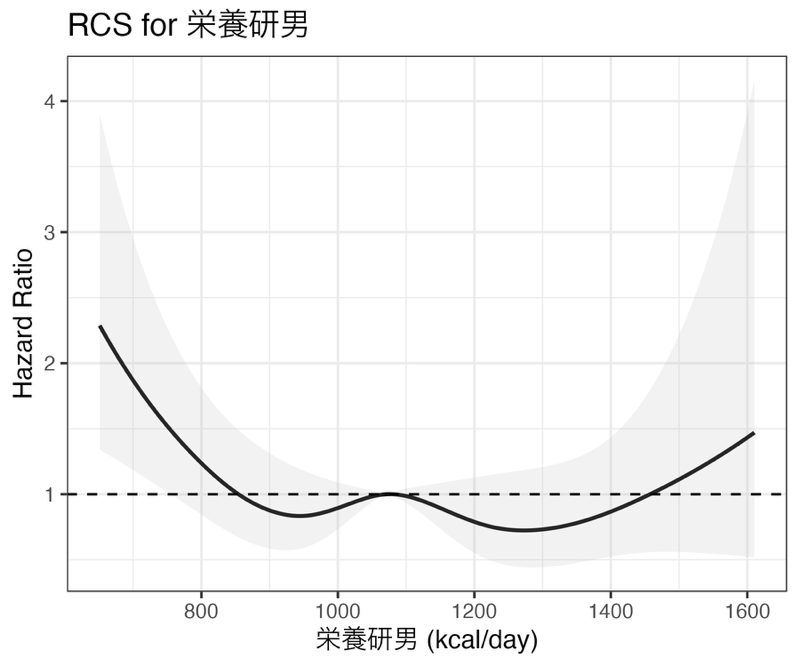


**NIBIOHN BMR**

**Cunningham BMR**


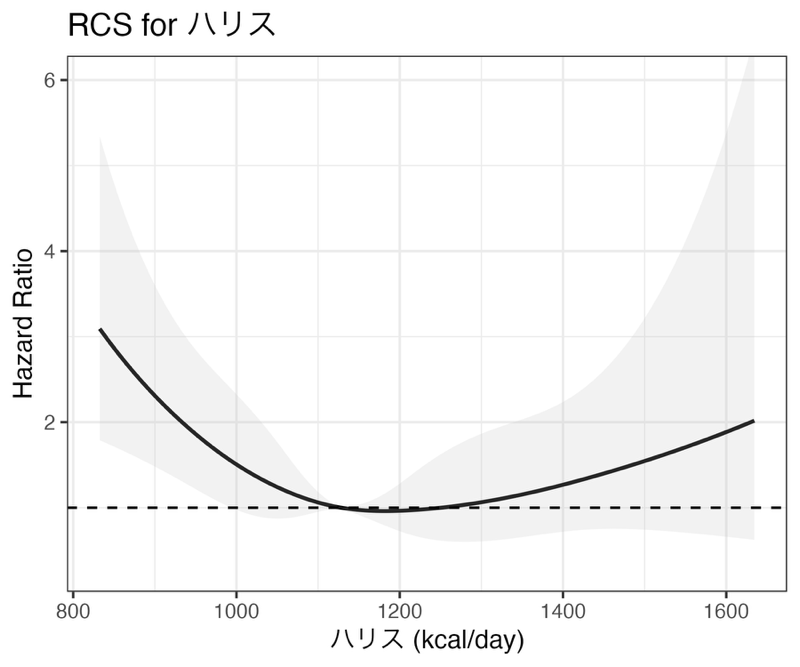


**Harris-Benedict BMR**


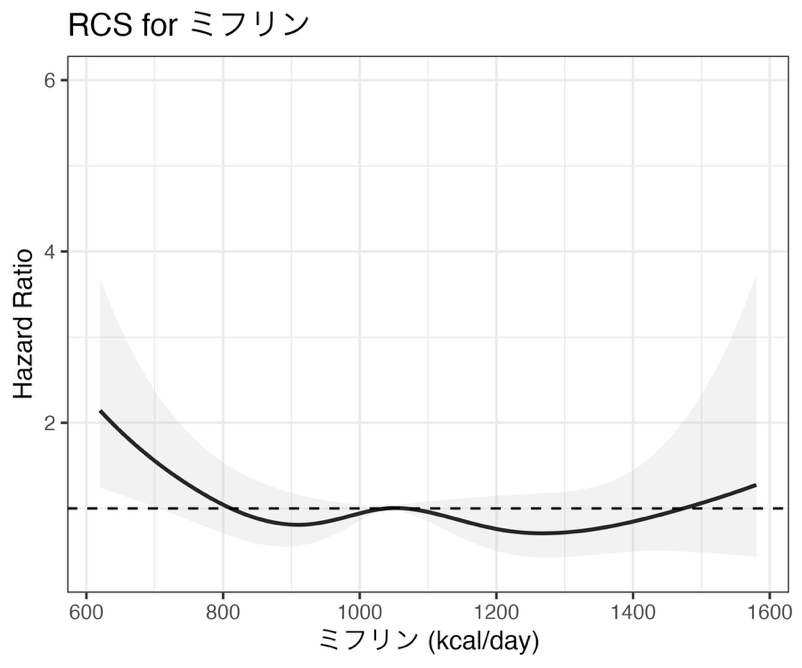


**Mifflin-St Jeor BMR**


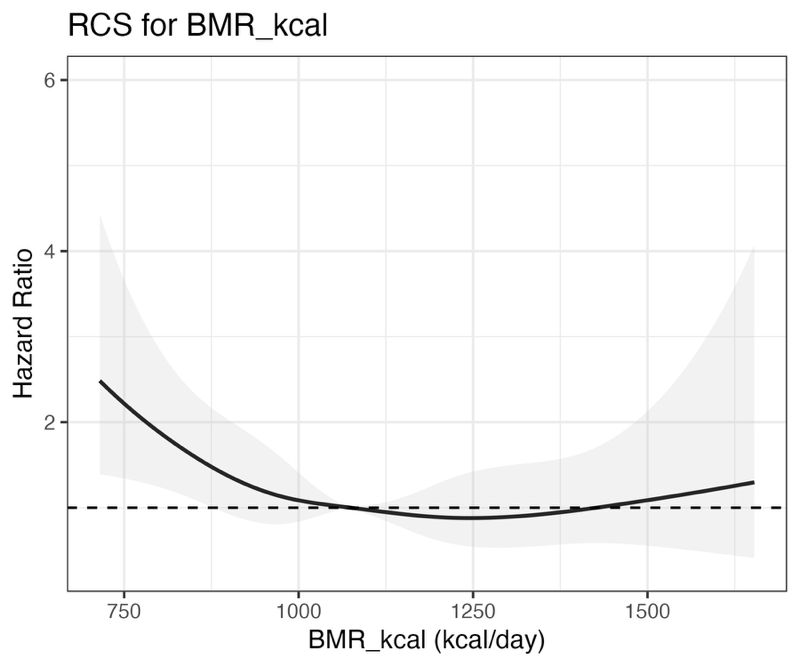


**TANITA BMR**

Supplemental Figure 2.
