# Supplementary material for: Basal metabolic rate predicts dementia in community-dwelling older adults: a 5-year longitudinal study
Source: Eur Geriatr Med. 2025 Oct 10;16(6):2181–91. doi: 10.1007/s41999-025-01322-9 (PMC12743684; doi:10.1007/s41999-025-01322-9)
Supplement: Supplementary file 3 — Fig. 3. Calibration plots for predicted dementia risk at 15, 30, 45, and 60 months using the Fine-Gray competing risk model. The dashed 45° line represents perfect agreement between predicted and observed risk. Solid lines indicate observed risks at each time point: 15 months (blue), 30 months (green), 45 months (orange), and 60 months (purple). (DOCX 755 KB) [file 41999_2025_1322_MOESM3_ESM.docx]

**NIBIOHN BMR**

**Cunningham BMR**

**Harris-Benedict BMR**

**TANITA BMR**

**Mifflin-St Jeor BMR**

Supplemental figure 2.
