# Supplementary material for: Basal metabolic rate predicts dementia in community-dwelling older adults: a 5-year longitudinal study
Source: Eur Geriatr Med. 2025 Oct 10;16(6):2181–91. doi: 10.1007/s41999-025-01322-9 (PMC12743684; doi:10.1007/s41999-025-01322-9)
Supplement: Supplementary file 4 — (DOCX 19 KB) [file 41999_2025_1322_MOESM4_ESM.docx]

Supplemental Table 1. Quartile cut points for each BMR formula and sex.

| Equation | Sex | 25th percentile | 50th percentile | 75th percentile |
| --- | --- | --- | --- | --- |
| TANITA [kcal/day] | All | 966 | 1074 | 1232 |
|  | Male | 1169 | 1273 | 1376 |
|  | Female | 923 | 992 | 1064 |
|  |  |  |  |  |
|  |  |  |  |  |
|  | Sex | 25th percentile | 50th percentile | 75th percentile |
| Harris-Benedict [kcal/day] | All | 1045 | 1128 | 1235 |
|  | Male | 1137 | 1244 | 1348 |
|  | Female | 1021 | 1083 | 1146 |
|  |  |  |  |  |
|  |  |  |  |  |
|  | Sex | 25th percentile | 50th percentile | 75th percentile |
| Mifflin-St Jeor [kcal/day] | All | 905 | 1038 | 1242 |
|  | Male | 1192 | 1279 | 1361 |
|  | Female | 849 | 928 | 1003 |
|  |  |  |  |  |
|  |  |  |  |  |
|  | Sex | 25th percentile | 50th percentile | 75th percentile |
| Cunningham [kcal/day] | All | 1239 | 1318 | 1509 |
|  | Male | 1461 | 1541 | 1618 |
|  | Female | 1252 | 1252 | 1301 |
|  |  |  |  |  |
|  |  |  |  |  |
|  | Sex | 25th percentile | 50th percentile | 75th percentile |
| NIBIOHN [kcal/day] | All | 935 | 1070 | 1249 |
|  | Male | 1196 | 1281 | 1370 |
|  | Female | 881 | 961 | 1039 |

**Note:** NIBIOHN; National Institute of Biomedical Innovation, Health, and Nutrition.
