# Supplementary material for: Basal metabolic rate predicts dementia in community-dwelling older adults: a 5-year longitudinal study
Source: Eur Geriatr Med. 2025 Oct 10;16(6):2181–91. doi: 10.1007/s41999-025-01322-9 (PMC12743684; doi:10.1007/s41999-025-01322-9)
Supplement: Supplementary file 6 — (DOCX 19 KB) [file 41999_2025_1322_MOESM6_ESM.docx]

Supplemental Table 3. Hazard ratios for the development of dementia for five different continuous variables (100 kcal/day).

| Equation | Sex | HR (95% CI) | p value |
| --- | --- | --- | --- |
| TANITA [ per 100 kcal/day] | All | 0.90 (0.75 – 0.97) | < 0.05 |
|  | Male | 0.93 (0.79 – 0.93) | < 0.05 |
|  | Female | 0.77 (0.64 – 0.92) | < 0.001 |
|  |  |  |  |
|  |  |  |  |
|  | Sex | HR (95% CI) | p value |
| Harris-Benedict [ per 100 kcal/day] | All | 0.92 (0.72 – 0.94) | < 0.05 |
|  | Male | 0.96 (0.82 – 0.96) | < 0.05 |
|  | Female | 0.76 (0.61 – 0.94) | < 0.001 |
|  |  |  |  |
|  |  |  |  |
|  | Sex | HR (95% CI) | p value |
| Mifflin-St Jeor [ per 100 kcal/day] | All | 0.93 (0.72 – 0.97) | < 0.05 |
|  | Male | 0.95 (0.77 – 1.178) | 0.072 |
|  | Female | 0.76 (0.64 – 0.91) | < 0.001 |
|  |  |  |  |
|  |  |  |  |
|  | Sex | HR (95% CI) | p value |
| Cunningham [ per 100 kcal/day] | All | 0.89 (0.72 – 0.94) | < 0.05 |
|  | Male | 0.90 (0.72 – 1.15) | 0.120 |
|  | Female | 0.67 (0.51 – 0.86) | < 0.001 |
|  |  |  |  |
|  |  |  |  |
|  | Sex | HR (95% CI) | p value |
| NIBIOHN [ per 100 kcal/day] | All | 0.92 (0.71 – 0.95) | < 0.05 |
|  | Male | 0.96 (0.79 – 1.06) | 0.089 |
|  | Female | 0.79 (0.67 – 0.93) | < 0.05 |

Note: Cox proportional hazards models were adjusted for age, sex, height, weight, gait speed, MMSE, GDS score, education, smoking, heart disease, hypertension, diabetes disease, and hyperlipidemia.

BMR; Basal Metabolic Rate, HR; Hazard Ratio, CI; Confidence Interval, NIBIOHN; National Institute of Biomedical Innovation, Health, and Nutrition.
