# Supplementary material for: Basal metabolic rate predicts dementia in community-dwelling older adults: a 5-year longitudinal study
Source: Eur Geriatr Med. 2025 Oct 10;16(6):2181–91. doi: 10.1007/s41999-025-01322-9 (PMC12743684; doi:10.1007/s41999-025-01322-9)
Supplement: Supplementary file 7 — (DOCX 22 KB) [file 41999_2025_1322_MOESM7_ESM.docx]

| Predictor | Quartile | SHR (95% CI) | p-value |
| --- | --- | --- | --- |
| TANITA BMR | Q4 | *Reference* |  |
|  | Q3 | 1.11 (1.06 – 1.18) | < 0.001 |
|  | Q2 | 1.12 (1.05 – 1.19) | < 0.001 |
|  | Q1 | 1.23 (1.13 – 1.34) | < 0.001 |
|  |  |  |  |
| Mifflin-St Jeor BMR | Q4 | *Reference* |  |
|  | Q3 | 1.14 (1.08 – 1.21) | < 0.001 |
|  | Q2 | 1.13 (1.06 – 1.21) | < 0.001 |
|  | Q1 | 1.29 (1.17 – 1.42) | < 0.001 |
|  |  |  |  |
| Harris-Benedict BMR | Q4 | *Reference* |  |
|  | Q3 | 1.14 (1.08 – 1.21) | < 0.001 |
|  | Q2 | 1.16 (1.09 – 1.23) | < 0.001 |
|  | Q1 | 1.25 (1.12 – 1.29) | < 0.001 |
|  |  |  |  |
| Cunningham BMR | Q4 | *Reference* |  |
|  | Q3 | 1.11 (1.05 – 1.17) | < 0.001 |
|  | Q2 | 1.11 (1.04 – 1.19) | < 0.001 |
|  | Q1 | 1.23 (1.11 – 1.35) | < 0.001 |
|  |  |  |  |
| NIBIOHN BMR | Q4 | *Reference* |  |
|  | Q3 | 1.13 (1.07 – 1.19) | < 0.001 |
|  | Q2 | 1.12 (1.05 – 1.2) | < 0.001 |
|  | Q1 | 1.24 (1.14 – 1.36) | < 0.001 |

Supplemental Table 4. Subdistribution Hazard Ratios for Dementia Incidence by Quartiles of Five Different Indicators (Full Model)

**Note:** Fine-Gray subdistribution hazard models were adjusted for age, sex, height, weight, gait speed, MMSE, GDS score, education, smoking, heart disease, hypertension, diabetes disease, and hyperlipidemia. The fourth quartile (Q4) was used as the reference category. BMR; Basal Metabolic Rate, SHR; Subdistribution Hazard Ratio, CI; Confidence Interval, NIBIOHN; National Institute of Biomedical Innovation, Health, and Nutrition.
