# Supplementary material for: Basal metabolic rate predicts dementia in community-dwelling older adults: a 5-year longitudinal study
Source: Eur Geriatr Med. 2025 Oct 10;16(6):2181–91. doi: 10.1007/s41999-025-01322-9 (PMC12743684; doi:10.1007/s41999-025-01322-9)
Supplement: Supplementary file 8 — (DOCX 20 KB) [file 41999_2025_1322_MOESM8_ESM.docx]

Supplemental Table 5. omparison of apparent and optimism-corrected time-dependent AUCs for dementia prediction

| **TANITA [kcal/day]** |  |  |  |
| --- | --- | --- | --- |
| **Time (months)** | **Apparent AUC** | **Optimism-corrected AUC** | **95% CI** |
| **15** | 0.72 | 0.70 | 0.65 - 0.78 |
| **30** | 0.69 | 0.69 | 0.64 - 0.74 |
| **45** | 0.66 | 0.63 | 0.57 - 0.71 |
| **60** | 0.66 | 0.62 | 0.58 – 0.69 |
|  |  |  |  |
| **Mifflin-St Jeor [kcal/day]** |  |  |  |
| **Time (months)** | **Apparent AUC** | **Optimism-corrected AUC** | **95% CI** |
| **15** | 0.74 | 0.70 | 0.70 – 0.80 |
| **30** | 0.70 | 0.70 | 0.65 – 0.74 |
| **45** | 0.67 | 0.62 | 0.63 – 0.70 |
| **60** | 0.67 | 0.59 | 0.63 – 0.70 |
|  |  |  |  |
| **Harris-Benedict [kcal/day]** |  |  |  |
| **Time (months)** | **Apparent AUC** | **Optimism-corrected AUC** | **95% CI** |
| **15** | 0.74 | 0.74 | 0.70 – 0.79 |
| **30** | 0.72 | 0.69 | 0.64 – 0.76 |
| **45** | 0.70 | 0.68 | 0.61 – 0.75 |
| **60** | 0.71 | 0.68 | 0.62 – 0.74 |
|  |  |  |  |
| **Cunningham [kcal/day]** |  |  |  |
| **Time (months)** | **Apparent AUC** | **Optimism-corrected AUC** | **95% CI** |
| **15** | 0.71 | 0.70 | 0.66 – 0.77 |
| **30** | 0.69 | 0.64 | 0.61 – 0.73 |
| **45** | 0.66 | 0.64 | 0.62 – 0.69 |
| **60** | 0.66 | 0.58 | 0.52 – 0.68 |
|  |  |  |  |
| **NIBIOHN [kcal/day]** |  |  |  |
| **Time (months)** | **Apparent AUC** | **Optimism-corrected AUC** | **95% CI** |
| **15** | 0.73 | 0.72 | 0.69 – 0.78 |
| **30** | 0.68 | 0.62 | 0.64 – 0.73 |
| **45** | 0.66 | 0.60 | 0.62 – 0.69 |
| **60** | 0.66 | 0.55 | 0.50 – 0.69 |

Note: Apparent AUC represents the time-dependent area under the curve calculated using the original dataset. Optimism-corrected AUC was obtained via 1,000 bootstrap resamples to adjust for overfitting. Higher AUC indicates better discrimination.

BMR; Basal Metabolic Rate, AUC; Area Under the Curve, CI; Confidence Interval, NIBIOHN; National Institutes of Biomedical Innovation, Health and Nutrition.
